# Brain Metastasis-Initiating Cells: Survival of the Fittest

**DOI:** 10.3390/ijms15059117

**Published:** 2014-05-22

**Authors:** Mohini Singh, Branavan Manoranjan, Sujeivan Mahendram, Nicole McFarlane, Chitra Venugopal, Sheila K. Singh

**Affiliations:** 1 McMaster Stem Cell and Cancer Research Institute, McMaster University, 1280 Main Street West, Hamilton, ON L8S 4K1, Canada; E-Mails: msingh0285@hotmail.com (M.S.); branavan.manoranjan@medportal.ca (B.M.); smahendram@gmail.com (S.M.); mcfarln@mcmaster.ca (N.M.); venugop@mcmaster.ca (C.V.); 2 Department of Biochemistry and Biomedical Sciences, Faculty of Health Sciences, McMaster University, 1280 Main Street West, Hamilton, ON L8S 4K1, Canada; 3 Department of Surgery, Faculty of Health Sciences, McMaster University, 1280 Main Street West, Hamilton, ON L8S 4K1, Canada

**Keywords:** brain metastasis, brain tumor, cancer stem cell, brain metastasis-initiating cell, brain tumor-initiating cell

## Abstract

Brain metastases (BMs) are the most common brain tumor in adults, developing in about 10% of adult cancer patients. It is not the incidence of BM that is alarming, but the poor patient prognosis. Even with aggressive treatments, median patient survival is only months. Despite the high rate of BM-associated mortality, very little research is conducted in this area. Lack of research and staggeringly low patient survival is indicative that a novel approach to BMs and their treatment is needed. The ability of a small subset of primary tumor cells to produce macrometastases is reminiscent of brain tumor-initiating cells (BTICs) or cancer stem cells (CSCs) hypothesized to form primary brain tumors. BTICs are considered stem cell-like due to their self-renewal and differentiation properties. Similar to the subset of cells forming metastases, BTICs are most often a rare subpopulation. Based on the functional definition of a TIC, cells capable of forming a BM could be considered to be brain metastasis-initiating cells (BMICs). These putative BMICs would not only have the ability to initiate tumor growth in a secondary niche, but also the machinery to escape the primary tumor, migrate through the circulation, and invade the neural niche.

## 1. Introduction

Advancements in the treatment of systemic diseases along with timely screening and imaging protocols have led to an increase in the overall and progression-free survival of primary malignancies. Unfortunately, for many of these patients who have won their battle with cancer, their war remains far from over as recent epidemiological studies have shown an increase in the incidence and prevalence of brain metastasis (BM) [1,2]. Metastases are the most common type of neoplasm to affect the adult central nervous system (CNS), and are associated with poor prognosis as well as significant morbidity [3]. BMs occur in up to 40% of cancer patients [4], representing an incidence that is ten times greater than that of primary brain tumors [5,6]. Aside from their frequency, the burden of BMs is further illustrated by a median survivorship of 1–2 months in palliative patients with multimodal therapy extending survival to only 4–6 months [7].

In contrast to their uniformly fatal prognosis, the localization, tissue-specific primary origin, and clinical presentation of BMs are all quite variable. While the majority of BMs are found in the cerebral hemispheres (80%), they may also be located in the cerebrum (15%) and brainstem (5%) [8]. Interestingly, unlike primary malignant gliomas, BMs maintain a well-delineated margin between the malignant and normal brain tissue, a characteristic that may be attributed to the cellular properties of the primary tumor [9]. Of the many primary malignancies, lung, breast, melanoma, renal, and colorectal cancers are the main sources for BM (Table 1), whereas other cancers such as prostate, liver, bladder, pancreatic, and uterine have a lower propensity to seed the brain [2,7,10]. Clinically, BMs may present in three distinct manners: metachronous, synchronous, and anachronous. The majority of patients are diagnosed with a BM following a known primary malignancy (metachronous). Less commonly, patients are diagnosed simultaneously with the primary tumor and BM (synchronous presentation), and rarely patients will be diagnosed with a BM prior to the detection of the primary cancer (anachronous presentation) [7]. Irrespective of the location, origin, and clinical presentation of BMs, current therapeutic efforts remain limited to multimodal approaches consisting of surgical resection, whole brain radiotherapy, stereotactic radiosurgery, and/or chemotherapy [1,9]. The lack of clinicallyor biologically-based targeted therapies is mainly due to the few conceptual frameworks and even fewer *in vitro* and *in vivo* model systems for studying BM. In this review we discuss recent evidence for the presence of brain metastasis-initiating cells (BMICs), which seed the brain and promote tumorigenesis. We focus on evidence from the metastatic process, the recent identification of brain tumor-initiating cells (BTICs), the presence of activated developmental signaling pathways in BMs, and how these cell-intrinsic pathways may promote tumor heterogeneity while presenting novel therapeutic targets.

## 2. The Metastatic Process

**Figure 1.**
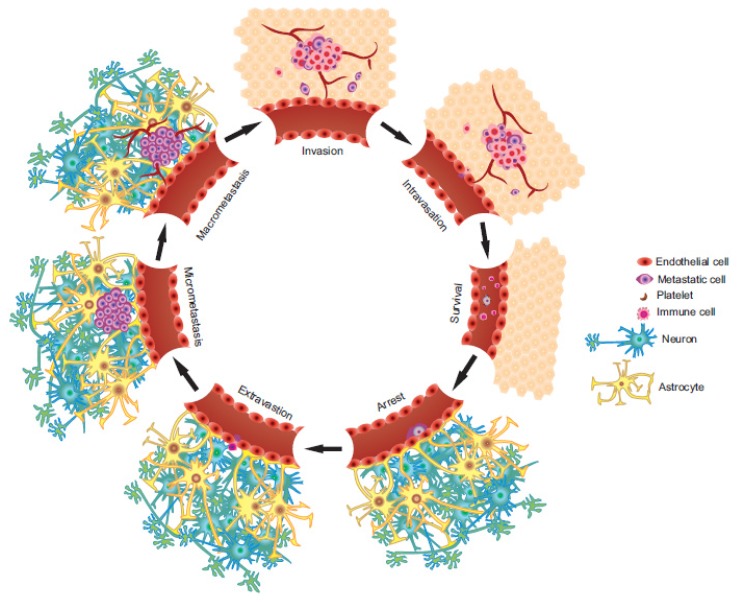
Stages of brain metastasis (BM). The general stages involved in the metastatic process.

The multistep metastatic cascade may be considered as a microcosm of Darwinian evolution in which survival of the fittest may be applied to a rare population of cells with the ability to endure the metastatic process. Albeit exceptionally intricate, metastasis is immensely inefficient with an estimated 0.01%–0.02% of cells shed from the primary tumor having the ability to establish metastatic growth at a secondary site [11–14]. The primary steps in metastasis (Figure 1) are further described below and include: invasion/migration, intravasation, circulation, arrest, extravasation, and survival in the secondary microenvironment.

### 2.1. Invasion/Migration

The ability of tumor cells to break away from the primary bulk tumor and invade surrounding tissue indicates the first stage of the multistep metastatic cascade. This step involves modifications to cell adhesion molecules as well as the extracellular matrix [15] with *E*-cadherin–catenin complexes serving as mediators of cell–cell adhesion, a critical aspect of the tumor cell cytoarchitecture. A switch in a tumor cell’s expression of cadherins initiates the secondary step of invasion, which promotes the dissociation of metastatic cells from the bulk tumor while facilitating their binding to the surrounding tissue [15,16]. Integrins also play a vital role in tumor cell migration and invasion by triggering multiple signaling transduction pathways to transmit signals in or out of the cell [17]. Furthermore, the interactions of receptor tyrosine kinases (RTKs) with integrins have been shown to stimulate the formation of a focal-adhesion kinase (Fak)-Src complex linked with many downstream cellular properties of invasion [15].

### 2.2. Intravasation

In order to gain access to distant sites in the body, tumor cells must intravasate into a venule, capillary, or lymphatic channel. This process is facilitated by tumor-associated macrophages and primary tumor cells, which secrete enzymes such as proteases responsible for degrading the basal membrane. Metastatic cells are then able to migrate through capillaries and lymph channels, resulting in their circulation within the venous system [18,19].

### 2.3. Circulation

Tumor cells within the circulatory system, termed circulating tumor cells (CTCs), must survive several lethal barriers. Shear forces encountered during circulation damage the majority of cells. In addition, the host’s own immune response, via natural killer cells, further sequester and destroy CTCs [20]. A characteristic feature of tumor cells is their anchorage-dependent growth; release from a substratum often results in anoikis, a detachment-induced apoptosis. This feature has been hypothesized to be a major contributor to metastatic inefficiency of tumor cells within the circulatory system and successful metastatic cells are capable of resisting anoikis by expressing multiple RTKs, invasion signaling components, and anti-apoptotic molecules [15,21]. Another feature of CTCs that promote their survival is their aggregation with other cellular elements such as fibrinogen, fibrin, and thrombin [1,15]. Platelets in particular enhance tumor cell survival in the circulation by forming a protective barrier around the cell, which protects against the immune response [22].

### 2.4. Arrest and Extravasation

As CTCs arrest within the brain vasculature, they undergo morphological changes suggestive of a transmigratory process through endothelial gaps, also known as diapedesis [23]. As a secondary tumor site, the brain itself possesses unique barriers for metastatic cells, specifically the lack of lymphatic drainage along with the blood–brain barrier (BBB) [24]. While the BBB functions as an initial gatekeeper, selectively permitting the entry of substances into the brain parenchyma, it also facilitates the growth and survival of metastatic tumor cells capable of circumventing the BBB. This is primarily achieved through preserving the immune-privileged nature of the brain along with preventing the entry of chemotherapeutic agents into the neural environment. It is interesting to note the propensity of some tumor types to metastasize to the brain despite the constraints of the BBB. Of the various primary malignancies, lung, breast, melanoma, renal, and colorectal cancers are the main sources for BM, whereas other cancers such as prostate, liver, bladder, pancreatic, and uterine do not have such a propensity to seed the brain [7,10,24].

### 2.5. Colonization of the Secondary Microenvironment

Once across the BBB the metastatic cells must now survive in the brain microenvironment, a process that leads to one of two outcomes: cell death or quiescence [25]. Tumor cell death is especially severe when arresting at the brain. Astrocytes react to extravasating tumor cells by secreting plasmin, which as opposed to its advantageous use during initial tumor invasion in early metastasis, actually prevents colonization of the brain parenchyma in two ways: (1) by activating the mobilization of FasL, a proapoptotic cytokine, to induce tumor cell apoptosis; and (2) by inactivating tumor cells expressing L1 cell adhesion molecule, L1CAM to inhibit their adhesion to brain capillaries [26]. The tumor cells combat this by secreting anti-PA (plasminogen activator) serine protease inhibitors (serpins), which inhibit the production of plasmin and consequently its effects [26,27]. Paradoxically, expression of various serpins by the host tissues and noninvasive carcinomas has been implicated in the inhibition of tumor progression [28–30].

Colonization of the brain requires the brain to provide a hospitable environment as well as the tumor cell being able to adapt to the neural environment, and it is this particular stage of metastasis that has been implicated in being one of the main barriers to BM formation [31]. The effects of the neural microenvironment have been shown in the case of melanoma, in which intracarotid injections of amelantoic melanoma cells formed a melantoic BM that reverted back into an amelantoic phenotype when transplanted subcutaneously out of the neural environment [32].

## 3. Frameworks for Studying Metastasis

Given the many primary and secondary organ sites that are encountered in metastatic cancers, an all-encompassing framework that accounts for the full spectrum of the metastatic cascade has proven to be exceedingly challenging. Interestingly, much of the contemporary literature on metastasis posits metastatic cells to display features reminiscent of traditional tumor-initiating cells (TICs), which is in keeping with the novel and emerging paradigm of the cancer stem cell (CSC) hypothesis. A review of previously established models further provides compelling evidence for not only the presence of metastatic stem cells but also their role in promoting BM.

### 3.1. Seed/Soil Hypothesis

The initial description of a research framework for the study of metastasis was proposed by Stephan Paget in 1889 [33]. Paget’s “seed and soil hypothesis” aimed to explain the mechanisms that drive metastatic cells to their ultimate location. According to the seed and soil hypothesis, BMs are not formed randomly (or stochastically) but may in fact be a consequence of the secondary nature of certain tumor cells—”seed”—that have a propensity for the neural environment [2,24,34]. Paget’s theory comprised three main principles: (1) a tumor is composed of a heterogeneous population of cells with different characteristics; (2) only certain cells possess the specific traits that allow them to metastasize; (3) formation of a secondary neoplasm depends on the interactions between the tumor cell “seed” and secondary site microenvironment “soil”. It is quite remarkable that even in the late 19th century, Paget was able to describe a model that attributes metastasis to a hierarchical cluster of cells with the metastatic-initiating cell at the apex of the hierarchy—the basis of the CSC hypothesis.

### 3.2. Mechanical Hypothesis

James Ewing’s “mechanical hypothesis” was proposed in 1928, four decades following Paget’s description of the seed and soil hypothesis [35]. The mechanical hypothesis attributes the circulatory system for the homing capacity of metastatic cells to their secondary site. Due to the larger size of cancer cells (approximately 20 μm) compared to that of an average vessel’s lumen (3–5 μm) [18], CTCs would be restricted to arresting in the first capillary bed of the initial organ they encounter [2,34,35]. Although the circulatory pattern is adequate to explain the location of certain metastases, this is not enough to corroborate the incidence of metastases to most secondary sites [34]. A review of clinical data on metastatic site predilections established that mechanical factors could account for metastases to secondary sites within the vicinity of the primary malignancy. However, this finding could not be reproduced in metastases to distant organs, which were subsequently determined to be driven by site-specific as opposed to mechanical/circulatory factors [36,37]. Furthermore, despite comparable blood flow, the liver is a much more common site of metastasis compared to the spleen. Abdominal and pelvic primary cancers tend to form BMs that far exceed the proportion as estimated from the blood supply [7]. As such, the circulation can only explain approximately 66% of metastatic cases [10], establishing a precedent for additional mechanisms of tumor seeding.

### 3.3. Epithelial–Mesenchymal Transition

Although epithelial–mesenchymal transition (EMT) [38] was first reported in 1908 to describe the reorganization of germinal layers [39], EMT has recently been reconceptualized as a cellular program that promotes the initial cellular invasion and intravasation of metastatic cells. EMT is a series of morphological and phenotypic changes that promote the conversion of epithelial and endothelial cells into a mesenchymal phenotype [24,40]. This occurs by way of the metastatic cell reducing the expression of adhesion molecules and intercellular junctions that tether it to neighboring cells (*E*-cadherin, adherens), while increasing the expression of mesenchymal-related molecules such as *N*-cadherin, fibronectin and vimentin. As a result, metastatic cells are able to dissociate from the primary bulk tumor and intravasate into the circulatory system. Upon entering the target organ, a secondary process known as mesenchymal–epithelial transition (MET) takes place. Similarly, the metastatic cells lose their mesenchymal traits and revert back to the original epithelial phenotype. While the molecular mechanisms underpinning both, EMT and MET have yet to be thoroughly elucidated [40], several elegant studies have demonstrated unique functional characteristics of cells capable of undergoing EMT as well as the role of EMT in the generation of CSCs [38,41–43]. Using colon carcinomas and their corresponding metastases, Brabletz and colleagues found that cells within the tumor core exhibited epithelial cell-like traits, whereas cells on the periphery expressed a more mesenchymal phenotype [44]. Morel *et al.* determined that activation of the Ras/MAPK signaling pathway in non tumorogenic mammary epithelial cells could generate a population expressing CD24^low^CD44^+^ stem cell like signatures displaying EMT characteristics [45]. shRNA-mediated knockdown of *E-cadherin* induced an EMT state in transformed HMLER breast cancer cells, and subsequently increased CD24^low^CD44^+^ populations with enhanced tumorsphere formation [46].

### 3.4. Cancer Stem Cell Hypothesis

The cancer stem cell (CSC) hypothesis suggests that a relatively small fraction of tumor cells termed, CSCs, have the ability to proliferate and maintain tumor growth [47]. This is in sharp contrast to all other cells of the bulk tumor, which are characterized by limited proliferative capacity and a more specified lineage potential. More specifically, a CSC maintains two key properties: self-renewal and multi-lineage differentiation. Self-renewal is defined as the ability of a parental cell to generate an identical daughter cell and a second cell of the same or different phenotype, whereas through the process of differentiation a CSC is able to give rise to the heterogeneous cell lineages that comprise the original tumor [47]. John Dick and colleagues were the first to provide evidence supporting the CSC hypothesis [48]. Upon performing limiting dilution assays of injecting leukemia cells into immunocompromised mice, they found that not only was tumor formation possible via one cell, but that the tumor recapitulated the original patient tumor heterogeneity. Following this, CSCs (also termed, tumor-initiating cells (TICs), and in the case of brain cancer, brain tumor-initiating cells (BTICs)) were also described in many solid tumors [49], such as the brain [50], breast [51], colon [52], and prostate [53,54]. Consequently, the CSC framework takes into account intratumoral heterogeneity by having a developmentally primitive cell at the apex of the hierarchy with a spectrum of more differentiated cells as one goes down this hierarchy [55].

**Figure 2.**
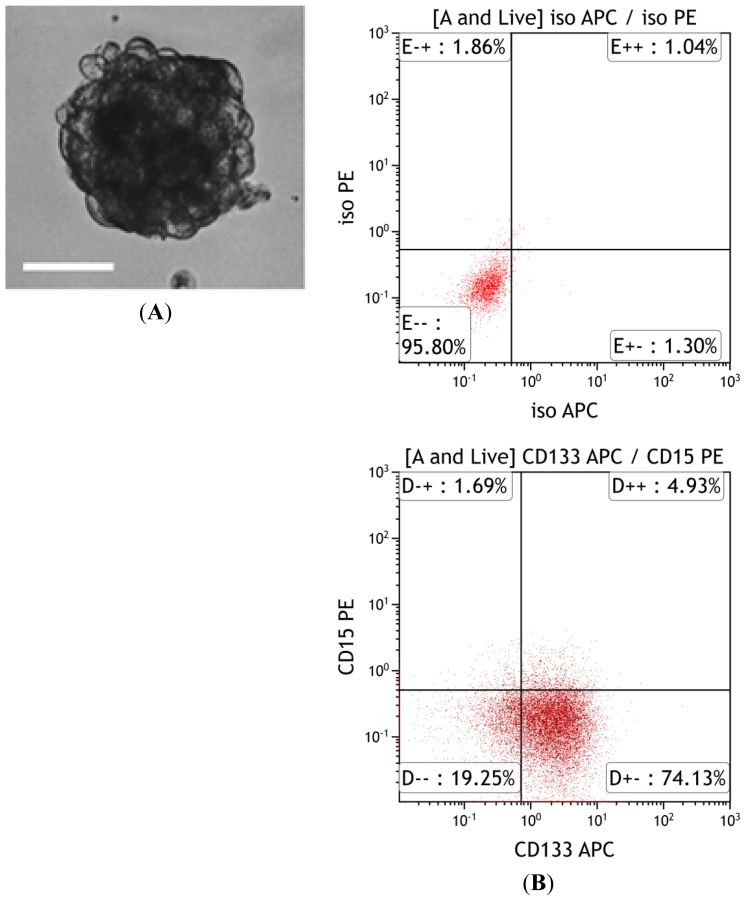
Characteristics of brain metastasis-initiating cells. (**A**) BM from the lung possess a cancer stem cells (CSCs) *in vitro* (100× magnification, 100 μm). Patient-derived BM samples were grown as tumorspheres in neural stem cell media; (**B**) CSC marker (*i.e.*, CD133 and CD15) expression assessed by flow cytometry.

Accomplishment of metastatic colonization could be influenced by an important property of migratory cells—a high self-renewal capacity in order to seed a large tumor. CSCs and in particular BTICs have been shown to survive lethal environmental pressures (hypoxia, low pH, nutrient deprivation, poor blood supply), evade host defenses (immune mediators), as well as circumvent growth suppressors and inhibitors of proliferation (cell cycle checkpoints, DNA damage control pathways) [2]. These cells are also able to bypass apoptosis by increasing expression of various antiapoptotic regulators and survival signals. Furthermore, quiescence, a feature that is often attributed to stem cells is characterized by limited cell cycle activity. The occurrence of BM from primary breast and melanoma years to decades following treatment of the primary malignancy suggests the growth of a fairly quiescent metastatic cell population over several years [56]. Given that many of these properties are shared by metastatic cells as they cycle through the metastatic cascade, it is reasonable to propose the presence of metastasis-initiating cells and in the case of brain tumors, brain metastasis-initiating cells (BMICs). Through the examination of epithelial and mesenchymal subtypes of prostate and bladder cancer cell lines, Terrassa *et al.* revealed that the more epithelial-like subpopulations had enhanced tumorsphere forming capacities and tissue colonization abilities when compared to mesenchymal subtypes that exhibited greater invasive properties [57]. The group concluded that in some cancers a tumor cell will acquire more invasive (mesenchymal) traits at the expense of its self-renewal (epithelial) properties, suggestive of a transient EMT process that may enhance the metastatic potential of a tumor. Expression of EMT-promoting transcription factors, such as Snail, Twist, and ZEB1 that were initially characterized in cancer cell invasiveness [38], have also been shown to promote entrance of the metastatic cells into the TIC state. Nolte *et al.* most recently reported the identification and purification of a subpopulation of cells from human BMs from primary lung cancer that had a marked capacity for proliferation, self-renewal, and differentiation, indicative of a BMIC population [58]. They demonstrated that BMs have a similar sphere-formation capacity as primary brain tumor samples when exposed to neural stem cell (NSC) conditions, where sphere-formation correlates with self-renewal. Known BTIC markers were also expressed in BMs, in percentages similar to those in primary brain tumors (Figure 2).

### 3.5. In Vivo Validation of Tumor-Initiating Capacity

While major advances in *in vitro* models have emerged to investigate the metastatic process, these models are only able to study specific steps of this intricate process. Many researchers utilize experimental animal models to assess the cellular and molecular interactions that occur during the extravasation step of the metastatic cascade of tumor cells into the brain. A successful *in vivo* model is defined by the injection of cells consistently migrating to the brain to form solid tumors. Such approaches have been established from lung, melanoma, and breast carcinomas to study BM [5]. Unfortunately, these models are still unable to fully recapitulate the entire metastatic process seen in patients with BM, though they still provide much insight into the specific mechanisms involved. The current BM framework can be divided into rodent syngeneic and human-rodent xenotransplantation groups. These models can be further subdivided based on the inoculation route of either ectopic or orthotopic, where the site of injection will greatly define the site of metastases. The preparation of the tumor cells themselves prior to injection can alter the formation of metastases *in vivo* [1]. The enzymatic solutions often utilized to harvest cells in culture can alter the expression of cell surface molecules, some of which may be involved in enabling tumor cell arrest. Specifically trypsin, a serine protease used to cleave cells growing adherently, has been shown to modify the direction of metastases [2,59].

Human-rodent xenograft models have been in development since the early 1970s using cancer-derived human tissue or cell lines into immunocompromised or immunocompatible rodents. Despite the limitation of using mice with an incomplete immune system, these models are useful in the examination of the associations between the microenvironment and human tumor cell in establishing metastases. Rodent syngeneic approaches are developed from the injection of murine derived cell lines into immunocompromised or immunocompatible mice. These are adequate models to examine metastatic dissemination of tumor cells and subsequent organ colonization, and are advantageous in that the transplanted tissue or cells, the microenvironment, and the host are all of the same species. However, these methods are restricted to studying only mouse cancer cell metastases and lack the specific features of human cancer, such as genetic complexity [60,61]. Spontaneous metastasis models are often used to overcome the limitations of experimental frameworks [1]. Although these models lack spontaneous BM development, the resulting tumors have provided several cell lines, which have subsequently been used to study BM [62]. Repeated intracardiac injections of these cells and the collection and reinjection of the subsequent but rare brain tumors formed led to the selection of two neurotropic cells lines that are predisposed to forming BMs exclusively [63]. These models are advantageous in the study of interactions and neural homing tendencies between BMICs and immune-competent host.

Ectopic/intravenous injections place cells directly into the blood stream, commonly either via intracardiac, intracarotid, or tail vein routes, and the inoculation site can influence where a tumor cell will extravasate. Ectopic injections are advantageous in that they permit the delivery of a controlled number of cells as well as the short time course required to form BMs. Zhang *et al.* injected Lewis lung carcinoma cells into the right internal carotid artery of C57BL/6NCrj mice and found metastatic cells in the left hemicerebrum after only 12 days, and were able to replicate the general process of lung cancer metastasis to the brain [64]. Unfortunately, none of the current ectopic injection routes completely reflect the nature of metastatic disease in humans as they bypass the initial steps of the metastatic cascade, possibly permitting the growth of tumor cells that would otherwise not be capable of intravasating into circulation [65].

Orthotopic injection is the process of injecting cells into the same organ of a mouse as the organ the cells originated from, and typically form metastases after a slow induction period. Orthotopic inoculation has led to the development of several BM models from various primary cancers. Mathieu and colleagues obtained 61% rate of BM formation through lung engraftment of A549 NSCLC cells followed by treatment with various chemotherapeutic agents [66]. Bos *et al.* performed serial *in vivo* selection of CN34 tumor cells from a breast cancer patient as well as MDA-MB-231 cell line in order to isolate populations that preferentially metastasize to the brain [67]. Orthotopic models face a huge limitation in that BM occurrence can be rare depending on the primary source. It remains undetermined if this is due to the BBB or the rapid primary tumor growth leading to death prior to the formation or identification of BMs. Intracranial injections are a more direct method to deliver cells isolated from BM to the brain parenchyma, though unfortunately this circumvents a majority of the metastatic stages and may allow the growth of cells that otherwise would not survive the metastatic process [65]. As such, this model is considered to reflect local growth rather than the capacity of cells to metastasize. Nolte *et al.* established the identification of CSCs in BMs through intracranial xenotransplantation of brain metastases from lung primary cancers [58].

### 3.6. Potential Therapeutic Targets in Brain Metastasis-Initiating Cells

There are several characteristics of BM and the metastatic process that could prove to be potential therapeutic targets. Several studies have identified a possible homing mechanism in BM, where secondary organs produce chemoattractants to draw in metastatic cells [18]. The most well-studied chemokine involved in BM is the CXC-chemokine ligand 12 (CXCL12 or SDF-1). This chemokine is secreted by stromal cells of various organs, and is thought to attract cells expressing its corresponding receptors CXCR4 and CXCR7. In the nervous system, expression of CXC12 is vital for neuronal guidance in the developing brain [68], while in cancer it functions by encouraging tumor cell extravastion, migration, and adhesion in the corresponding stromal environment. CXCL12 expression in tumor endothelial cells [69] and CXCR4 expression on metastatic tumors [70] were found to correlate with shorter patient survival, indicative of a more aggressive tumor group. CXCR4–CXCL12 interactions and downstream signaling enhance the proliferation and survival of tumor cells in distant inhospitable microenvironments [71,72], and have been implicated as crucial elements in the progression of BM [73,74]. Though the chemokine/receptor system requires further investigation in the BMIC population, these cells may exploit the chemoattractant gradient in order to home to the brain. As such targeting of CXCR4 expression on BMICs may be a potential option for limiting BM formation in some cancers, with a focus on expression modulation as studies have shown antagonism of CXCR4 may enhance a tumor cell’s metastatic ability [75].

Another molecule implicated in the homing mechanism of BM is CD44. CD44 is a glycosaminoglycan that is a major component of the neural environment, and is a specific receptor for hyaluronic acid [76,77]. It is a well characterized CSC marker in many cancers such as breast and prostate, and has been correlated with a more invasive phenotype [78]. Several studies have shown that targeting of CD44 and CD44-hyaluronan interactions at various levels can disrupt metastasis. Direct targeting of CD44 epitopes via antibodies has been seen to diminish metastatic properties of pancreatic cancer cells [79]. Hyaluronan-conjugated drugs target CD44’s ability to internalize hyaluronan, where the conjugated drug is released within CD44 and activated by enzymatic hydrolysis [80]. Though CD44 expression has yet to be confirmed in a BMIC population, the current knowledge of its high expression in brain metastases may implicate CD44 as a target to obstruct the ability of BMICs to home to the brain, subsequently limiting their invasion to the immediate environment of the primary cancer.

Several studies have undertaken the effort in identifying genes essential to BMs. Using genomic expression analysis, Bos *et al.* identified several genes that mediate breast cancer cell infiltration of the BBB [67]. One gene in particular, α-2,6-sialyltransferase (ST6GALNAC5), specifically mediates breast cancer metastasis to the brain by enhancing tumor cell adhesion to neural endothelial cell walls. Similar work by Nolte *et al.* identified 30 candidate genes as being significantly over-expressed in a stem cell population for BM, primary brain and lung tumors, 11 of which were found to be significant predictors of patient outcome [58]. Okuda *et al.* identified high expression of Kruppel-like factor 4 (KLF4) in CSCs of breast cancer, and upregulation of miR-7 was able to attenuate brain metastases [81]. Further characterization of these genes is required to identify ones vital to the function of BMICs.

Several pathways have been shown to support self renewal and metastatic potential of BMICs [2]. Xin *et al.* reported that increased levels of jagged 1 (JAG1) in astrocytes and subsequent interaction of metastatic breast CSCs with astrocytes was found to activate Notch signaling and promote metastasis formation and self-renewal of the metastatic CSCs in a neural environment [82]. Astrocytes have also been shown to indirectly activate the ERK1/2 pathway and increase MMP-2 expression, promoting invasion and formation of brain metastases in a rat adenocarcinoma [83]. Additionally, activation of WNT/TCF signaling, mediated through lymphoid-enhancer-binding factor (LEF1) and homeobox-leucine zipper protein (HOX9), has also been associated with enhanced brain colonization of lung adenocarcinoma cells [84]. Therapeutic efforts by way of inhibitors that target these pathways and/or their related components have been identified to impede brain metastases formation, such as the gamma secretase inhibitor, DAPT, for Notch and the natural MMP-2 inhibitor TIMP-2 [82]. Nevertheless, additional work is required to elucidate the specificity of these inhibitors in targeting the BMIC population.

An alternative target is the adaptation ability of BM to the neural environment to aid in colonization. In their exploration of BM from breast primary cancer, Newman *et al.* found the metastases to resemble a neural phenotype by exhibiting an over-expression of several γ-aminobutyric acid (GABA) variable traits such as GABA_A_ receptors, GABA transporters, and GABA transaminase [85]. The perturbation of the tumor microenvironment is a property that could be exploited in therapy development, by inhibiting the adaptation of BMICs to the neural environment and possibly their colonization of the neural environment.

The identification of genes, pathways, and markers vital to the function of BMICs and the formation of BMs may prove to be ideal therapeutic targets. Successful development of targeted therapies would lead to the arrest of metastasis to the brain, keeping the primary cancer in a localized state, and ultimately increasing patient survival. However, several challenges exist in selectively targeting the BMICs. The first of which is that BMICs account for a rare subgroup of cells within the bulk tumor and therefore only present at low frequencies [58]. Overcoming this limitation has led to adaptations and advancements to *in vitro* assays such as protein quantification and cell sorting in order to accurately characterize this rare cell fraction. Another significant drawback when studying BMICs is the lack of appropriate markers. Even if a dependable marker is found, the ability of BMICs to undergo asymmetric division suggests that a population enriched to homogeneity will eventually dilute itself out. This also leads to a third challenge of the depth of intratumoral heterogeneity; clonal evolution within the solid tumor could result in the brain metastasis having little or no resemblance to the primary tumor [86]. Establishment of temporal and spatial hierarchies of BMIC clones through clonal analysis and lineage tracing may be utilized to identify functionally distinct clones responsible for BM development.

## 4. Conclusions

The study of metastasis has drastically evolved over the past 200 years, with several key discoveries having been made in only the past decade. With the advent of novel *in vitro* assays and *in vivo* model systems, the identification of biological signaling mechanisms associated with the metastatic cascade have begun to emerge. Given the urgent desire for targeted therapies and the observation of a heterogeneous cellular landscape, other frameworks and model systems should be investigated for exploring the dynamic nature of metastasis. One such model system is that of the cancer stem cell (CSC) or brain metastasis-initiating cell (BMIC). The CSC model provides a framework to study the interplay between BMICs and their tumor niche, offering researchers with multiple perspectives regarding tumor biology and differential gene expression patterns in specific subsets of tumor cells. Although the CSC model provides several advantages in studying tumor heterogeneity, one must not neglect the limitations accompanied with this framework. These challenges primarily surround our ability to characterize these rare clonal populations of cells, which is particularly true for heterogeneous tumors such as metastases. Nevertheless, despite all of these shortcomings, the research in brain metastasis has grown at an admirable pace making the leap from the laboratory bench to patient bedside a realistic and foreseeable reality.

## Figures and Tables

**Table 1.** Common primary organ sources of brain metastasis.

Primary Source	Incidence of BM	Metastastic Features
Lung	40%–50%	Multiple metastatic lesions in the brain parenchyma in the early stages of the disease, and are associated with surrounding vasogenic edema
Breast	15%–25%	Single lesions found in the parenchyma and leptomeninges, with rare occurrences of vasogenic edema
Skin (Melanoma)	6%–11%	Multiple lesions form in the cortex as opposed to the grey-white junction, associated with hemorrhage
Colorectal	3%	Lesions in the supratentorial and cerebellar regions
Unknown	16%	Variable
